# Polyphenol Intake During Pregnancy and Childhood Asthma and Atopic Disorders: A Systematic Review

**DOI:** 10.1111/cea.70031

**Published:** 2025-03-12

**Authors:** Rachel J. Morgan, Cassidy Du Berry, Martin O'Hely, Lawrence Gray, Sarath Ranganathan, Peter Vuillermin, Peter D. Sly

**Affiliations:** ^1^ Institute of Mental and Physical Health and Clinical Translation Deakin University Geelong Australia; ^2^ Barwon Health Geelong Australia; ^3^ Respiratory Group Murdoch Children's Research Institute Melbourne Australia; ^4^ Department of Paediatrics University of Melbourne Melbourne Australia; ^5^ Department of Respiratory and Sleep Medicine The Royal Children's Hospital Melbourne Australia; ^6^ Respiratory Research@Alfred Monash University Melbourne Australia; ^7^ Child Health Research Centre The University of Queensland Brisbane Australia

**Keywords:** asthma, atopic diseases, children, flavonoids, polyphenols, pregnancy

**Summary**

- Insufficient evidence to determine if prenatal polyphenol intake affects prospective childhood asthma or atopy risk.
- Limited evidence suggests that polyphenol intake in children may improve asthma and atopy management.

To the Editor,

Asthma and atopic disorders, such as atopic dermatitis, food allergy and allergic rhinoconjunctivitis, significantly impact childhood well‐being and have a considerable health economic burden [1]. These conditions often co‐occur and progress in a pattern known as the ‘atopic march’, beginning with atopic dermatitis in infancy, followed by food allergy, and leading to wheeze and asthma in later childhood [2]. Several systematic reviews have highlighted maternal and childhood nutrition as a modifiable factor in the prevention and management of asthma and atopic disorders, yet there is limited evidence exploring the dietary component polyphenols in this association. Polyphenols are naturally occurring plant compounds with pre‐clinical evidence demonstrating their protective effect in the development of asthma and atopic diseases through antioxidant and anti‐inflammatory pathways [3].

We conducted a systematic review to examine the relationship between polyphenol intake during pregnancy and childhood and the prospective risk and management of asthma and atopic disorders in children. In January 2024, we searched three electronic databases: Scopus, EMBASE and MEDLINE. We included quantitative studies that recorded or supplemented polyphenol intake in pregnant women or children (aged 0–18 years). The primary outcome in prenatal studies was the prospective diagnosis of asthma or atopic disease in childhood, and in studies set in childhood, the management of asthma or atopic disease. Exclusion criteria were non‐English language articles, adult participants (≥ 18 years) and studies where the polyphenol content of the intervention or observed intake was not measured. Quality assessment used the Risk‐of‐bias tool (Rob2) for randomised controlled trials and ROBINS‐I and ROBINS‐E tools for non‐randomised and observational studies. The certainty of the evidence was assessed using the GRADE approach (PROSPERO ID: 42023495048). From the initial 294 studies, 46 were retrieved following abstract screening, and after further exclusions, six studies were included; their general characteristics are summarised in Table 1.

**TABLE 1:** Key characteristics of studies exploring the effects of polyphenol intake on atopic disease outcomes.

Author	Study design	Included participants	Dietary polyphenol intervention/observation	Atopic disease outcome	Outcome measures	Results	Risk‐of‐bias
Ghozal 2023 [4]	Cohort study	1324 Mother–child pairs followed up to 8 years	Observation: Concentrations of chemicals assessed in food items from food frequency surveys Polyphenols assessed: Biochanin‐A, Coumestrol, Daidzein, Enterolactone, Equol, Formononetin, Genistein, Glycitein, Matairesinol, Resveratrol, Secoisolariciresinol	AD FA AR Wheeze Asthma	Parental reported questionnaires assessing AD, FA, AR, wheeze and asthma	Intake of resveratrol associated with lower prospective risk of AR (aOR 95% CI: 0.73, 0.55–0.97, *p* = 0.03, *q* < 0.1) Intake of matairesinol associated with lower risk of asthma (aOR 95% CI: 0.85, 0.73–0.99, *p* = 0.03, *q* > 0.1) No results reported for association with polyphenols and FA or AD	Very high risk Selective exposure reporting following multiple exposure measurements Tool: ROBINS‐E
Sakihara 2022 [5]	Retrospective cohort study of a RCT	235 newborns followed up to 6 months	Dietary plan: Recorded Soy formula (Soy isoflavone) intake > 14 days per month and > 1350 mL per month between 1 and 2 months of age	Food Sensitisation	Skin prick test (3 mm or larger wheal diameter response)	Regular soy formula intake had a protective effect against development of food sensitisation (aOR 95% CI: 0.32, 0.16–0.62, *p* = 0.0007) at 6 months	High risk No details on management of missing data Tool: ROBINS‐E
Kojima 2000 [6]	Pilot: Non‐randomised experimental study	24 children with AD (mean age 13.3 years)	Intervention: 10 mg/kg of ACT daily for 10 weeks	AD	Skin scores: 0–2 in ascending order of severity at the face, trunk, arms and legs (/24) Scores for itching and sleep disturbance: 0–3 in ascending order of severity Total score/30	In the ACT group total, inflammation, lichenification and cracking scores decreased from baseline. Itching and sleep disturbance scores in the ACT group decreased more rapidly compared with the control group No between‐group analyses performed	Moderate risk Measurement of outcomes may be influenced by knowledge of intervention Tool: ROBINS‐I
Marseglia 2019 [7]	RCT	146 children with AR (mean age 9.1 years)	Intervention: 150 mg of Quercetin, 80 mg of *Perilla frutescens* (Rosmarinic acid, luteolin, apigenin, chrysoeriol) daily for 4 weeks	AR	Total Symptoms Score Overall symptom control (diary data) Quality of life questionnaire	Decrease in Total Symptom Score for intervention group (63.6%) and placebo group (60.7%) from baseline. There was no significant between‐group difference for total symptoms scores	Some concerns No details of prespecified data analysis plan Tool: RoB 2
Marseglia 2019 [8]	RCT	146 children with AR (mean age 9.1 years)	Intervention: 150 mg of Quercetin, 80 mg of *Perilla frutescens* (Rosmarinic acid, luteolin, apigenin, chrysoeriol) daily for 12 weeks	AR	Symptom‐free days AR exacerbation Cumulative days treated with rescue medication	The intervention group had more symptom‐free days (HR 95% CI: 0.54, 0.29–0.99, *p* = 0.04), less exacerbations (*p* = 0.04) and lower number of days requiring rescue mediation (*p* < 0.0001)	High risk No blinding to intervention Tool: RoB 2
Lau 2004 [9]	RCT	60 children (6–18 years) with mild‐to‐moderate asthma	Intervention: 1 mg/lb. tablet of Pycnogenol (formed of catechin, epicatechin, taxifolin, procyanidins and proanthocyanidins) daily for 12 weeks	Asthma	Lung function via peak flow meter Albuterol requirement Median Symptom Score	In the intervention compared to baseline, peak expiratory flow increased (*p* < 0.01), Albuterol inhaler use decreased (*p* < 0.001), Symptom score decreased (*p* < 0.001). In the control group, the findings showed minimal change. There were no between‐group differences reported	Some concerns No details of pre‐specified data analysis plan Tool: RoB 2

Abbreviations: ACT, apple condensed tannins; AD, atopic dermatitis; aOR, adjusted odds ratio; AR, allergic rhinoconjunctivitis; CI, confidence interval; FA, food allergy; HR, hazard ratio; RCT, randomised controlled trial; RoB 2, Cochrane risk‐of‐bias tool for randomised trials (version 2); ROBINS‐E, Risk of bias in non‐randomised studies of exposure; ROBINS‐I, Risk‐of‐bias in non‐randomised studies of interventions tool.

A prenatal cohort study (*n* = 1428) retrospectively assessed third trimester intake of chemicals, including 11 phytoestrogens, a polyphenol subclass [4]. Children born to mothers with multiple dietary intakes of resveratrol had a lower associated risk of parent‐reported allergic rhinoconjunctivitis (adjusted odds ratio [aOR] 95% confidence interval [CI]: 0.73, 0.55–0.97, *p* = 0.03) [4]. They also found that prenatal multiple dietary exposure to matairesinol was associated with a lower risk of asthma (aOR 95% CI: 0.85, 0.73–0.99, *p* = 0.03), although the analysis did not survive multiple testing [4]. This study is at high risk of bias due to recall bias and selective reporting of multiple exposure measurements; therefore, these results should be interpreted with caution [4].

A longitudinal study of 235 infants showed regular soy isoflavone intake (≥ 14 days per month and ≥ 1350 mL per month) was associated with a decreased likelihood of food sensitivity (aOR 95% CI: 0.32, 0.16–0.62, *p* < 0.001), hen's egg sensitivity (aOR 95% CI: 0.42, 0.20–0.88, *p* = 0.02) and cow's milk sensitivity (aOR 95% CI: 0.33, 0.14–0.81, *p* = 0.02) [5]. The authors did not detail the management of missing data in the study, placing it at a high risk of bias [5].

Four studies investigated polyphenol supplementation in children with established asthma and atopic conditions [6, 7, 8, 9]. A study of 28 children with atopic dermatitis found that an intervention of 10 weeks of apple‐condensed tannins reduced total inflammation, lichenification and cracking scores [6]. However, the study lacked between‐group analyses and is at high risk of bias due to lack of concealment of allocation and intervention [6]. Two of the studies investigated supplementation with Lertal (containing Quercetin, *Perilla frutescens* and Vitamin D) for allergic rhinoconjunctivitis management [7, 8]. While the 4‐week randomised double‐blinded placebo‐controlled trial (*n* = 146) found no significant between‐group differences [7], the subsequent 12‐week open parallel‐group study (*n* = 128) showed significantly more symptom‐free days (Hazard Ratio, 95% CI: 0.54, 0.29–0.99, *p* < 0.05), fewer exacerbations (25% vs. 42%) and reduced rescue medication use (153 days vs. 683 days) in the intervention group compared to controls [8]. However, due to the lack of blinding in the 12‐week intervention, it was at a higher risk of bias [8]. A 12‐week trial of Pycnogenol (formed of catechin, epicatechin, taxifolin, procyanidins and proanthocyanidins) in children with asthma (*n* = 60) decreased the number of puffs of rescue medication per day from mean (SD): 2.57 (0.16) to 0.22 (0.07), *p* < 0.001, and decreased the score from mean (SD): 2.25 (0.13) to 0.27 (0.06), *p* < 0.001 [9]. The placebo group saw more modest improvements across these measures, but there were no between‐group differences reported [9].

Our systematic review found insufficient high‐quality evidence to support or refute whether polyphenol intake affects asthma and atopic outcomes in children. Studies investigating polyphenol intake in children were inconclusive, and while methodological flaws limited conclusions, evidence suggests polyphenol supplementation may improve asthma and atopic management. The heterogeneity in polyphenol measurements, outcome definitions and follow‐up periods hindered direct comparisons and precluded meta‐analysis. Additionally, there is a lack of studies quantifying maternal polyphenol consumption in relation to offspring asthma and atopic disease. Despite pre‐clinical evidence suggesting maternal polyphenols protect against asthma and atopic outcomes via gut microbiome interactions and downregulating proinflammatory pathways, human studies remain lacking.

Future research should prioritise well‐designed randomised controlled trials with standardised polyphenol measurements and validated outcome assessments. Particular attention should be given to pregnancy interventions, given the potential for early modification of disease trajectory. Should future research confirm the benefits of polyphenol intake, increasing polyphenol‐rich foods in the diets of pregnant women and children could offer an accessible strategy to reduce the risk of asthma and atopic diseases.

## Author Contributions

R.J.M., C.D.B., M.O., S.R., P.V. and P.D.S. contributed to study planning and protocol development. R.J.M., C.D.B. and P.D.S. contributed to the literature database search, collection and interpretation of data. All authors contributed to drafting the manuscript and approved the final draft of the manuscript submitted for publication.

## Conflicts of Interest

The authors declare no conflicts of interest.

## Data Availability

The data that support the findings of this study are openly available in Figshare at https://figshare.com/articles/figure/Polyphenol_intake_during_pregnancy_and_childhood_asthma_and_atopic_disorders_a_systematic_review/28502183 reference number 10.6084/m9.figshare.28502183.
